# A case report: Nonsecretory multiple myeloma presenting with bone pain

**DOI:** 10.1097/MD.0000000000036951

**Published:** 2023-02-02

**Authors:** Qianshuang Geng, Jie Li, Xi Li, Wenjie Zhang, Guoxiang Zhang, Li Ge, Li Liang

**Affiliations:** a Department of Hematology, Heping Hospital Affiliated to Changzhi Medical College, Changzhi, China; b Department of Oncology and Hematology, Liuyang Hospital of Traditional Chinese Medicine, Hunan University of Chinese Medicine, Changsha, China; c Department of Nephrology, Heping Hospital Affiliated to Changzhi Medical College, Changzhi, China; d Department of Image, Heping Hospital Affiliated to Changzhi Medical College, Changzhi, China.

**Keywords:** bone pain, free light chain, nonsecretory multiple myeloma

## Abstract

**Rationale:**

Nonsecretory multiple myeloma (NSMM) is a rare subtype of multiple myelom, occurring in 1% to 2% of multiple myelom and characterized by the inability of clonal plasma cells to synthesize or secrete immunoglobulins. We describe a 71-year-old male patient who began with bone pain and was referred to hospital several times, but was not properly diagnosed and effectively treated.

**Patient concerns:**

A 71-year-old male patient visited our hematology department, complaining of lumbago for 1 year and back pain for half a year.

**Diagnoses:**

Low-dose whole-body bone computed tomography: multiple bone destruction of the sternum, ribs, multiple vertebrae and accessories of the spine, pelvis, bilateral humerus, and proximal femur. Monoclonal plasma cells accounted for 17.5% of nuclear cells in bone marrow puncture smear. Fluorescence in situ hybridization detected amplification of CKS1B (1q21) gene. Immunofixation electrophoresis negative. About 10.72% of monoclonal plasma cells were detected by flow cytometry. Finally, he was diagnosed with NSMM.

**Interventions:**

The patients received VCD chemotherapy (bortezomib 1.3 mg/m^2^, d1, d4, d8, d11; cyclophosphamide 300 mg/m^2^, d1–2, d8–9; dexamethasone sodium phosphate 20 mg, d1–2, d4–5, d8–9, d11–12, once every 21 days).

**Outcomes:**

After 2 cycles of VCD treatment, the symptoms of bone pain were significantly relieved, and the efficacy was evaluated as partial response. Follow-up chemotherapy will continue to be completed on schedule. We will continue to follow up to further evaluate the overall survival and progression-free survival.

**Lessons:**

This case shows that NSMM is easily missed or misdiagnosed.

## 1. Introduction

Multiple myelom is a disease that abnormal proliferation of monoclonal plasma cells in bone marrow, which is the second most common malignancy in the hematological system.^[1]^ Characterized by plasma cells in bone marrow to produce a large number of monoclonal immunoglobulin, deposition in the tissues or organs, causing high viscosity syndrome, amyloidosis, marrow infiltration, bone lesions, renal dysfunction and a series of pathological changes, the common clinical manifestations of “CRAB” symptoms (calcium elevation, renal insufficiency, anemia, bone disease).^[2]^ According to the type of M protein, MM can be divided into: IgG, IgA, IgD, IgM, and IgE type, light chain, double clone, and nonsecretory type.

Nonsecretory multiple myeloma (NSMM) is a rare subtype of MM that has more than 10% monoclonal plasma cells in the bone marrow but does not produce or secrete immunoglobulins. Serum and urine immunofixation electrophoresis monoclonal immunoglobulins and free light chain (FLC) tests are negative.^[3]^ Clinically, they are often treated for bone pain without obvious anemia, hypercalcemia and abnormal renal function in the early stage, which is easy to be ignored by doctors.

Therefore, we describe a case and share its diagnosis and treatment to provide clinical experience for better understanding of NSMM.

## 2. Case presentation

Our patient is a 71-year-old man with a history of hypertension and type 2 diabetic mellitus. No special family history. Since 1 year ago, he began to complain of low back pain and visited the department of orthopedics and pain medicine for several times. Computed tomography (CT) and MRI examinations were performed, indicating multiple bone destruction. Osteoporosis, lumbar disc herniation and fracture were diagnosed successively, and acupuncture, cupping, small needle knife, and bone cement injection were given for treatment, but the pain was not effectively controlled. In the past year, the low back pain continued to worsen and expanded into the whole back pain. CT showed multiple bone destruction, blood routine examination showed anemia, low dose CT of whole body bone revealed multiple bone destruction, further bone marrow puncture examination showed plasma cells exceeding 10%, serum immunoglobulin decreased, hematuria immunofixation electrophoresis and FLC negative, and finally confirmed NSMM. Examined patients with anemia, sternal tenderness is positive, superficial lymph node untouched enlargement.

**Figure 1.**
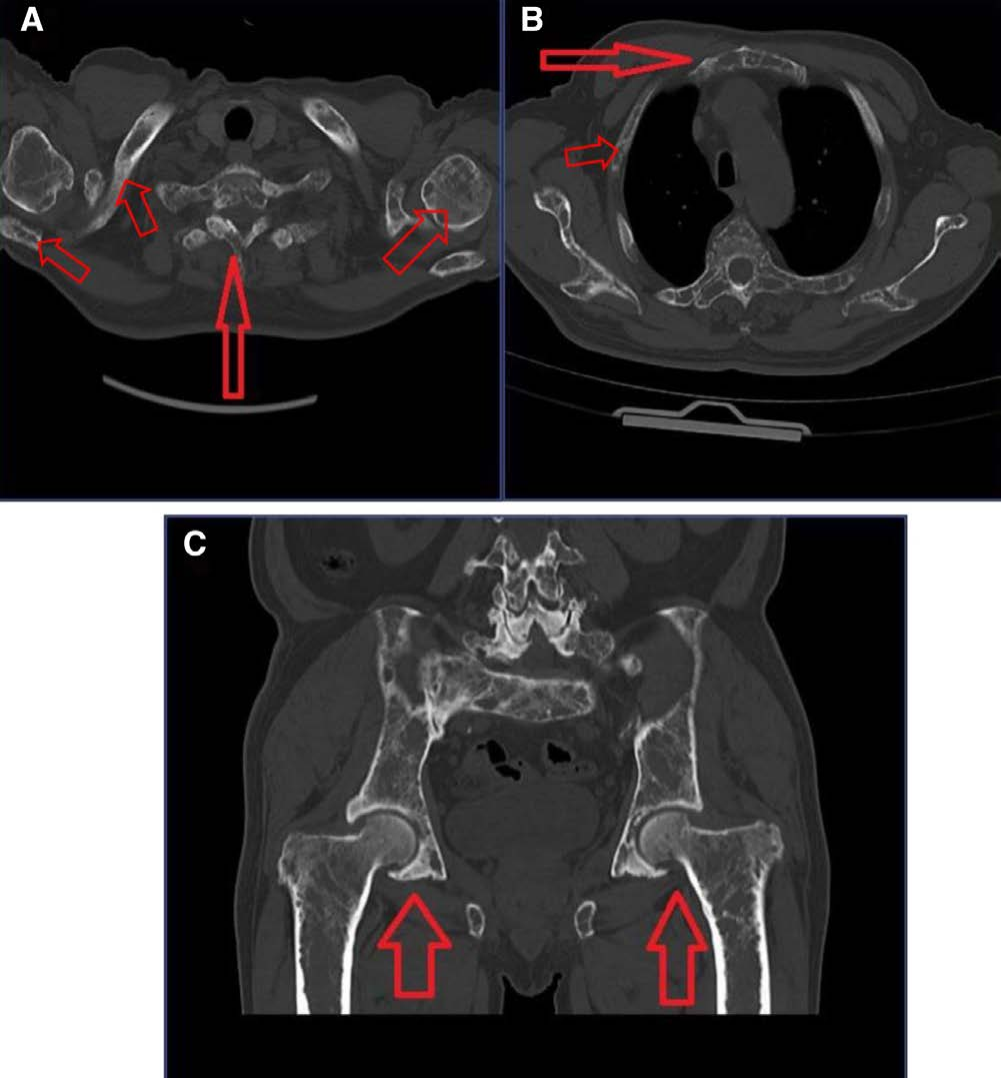
Low-dose whole-body bone CT: multiple bone destruction of the sternum, ribs, multiple vertebrae and accessories of the spine, pelvis, bilateral humerus and proximal femur. CT = computed tomography.

**Figure 2.**
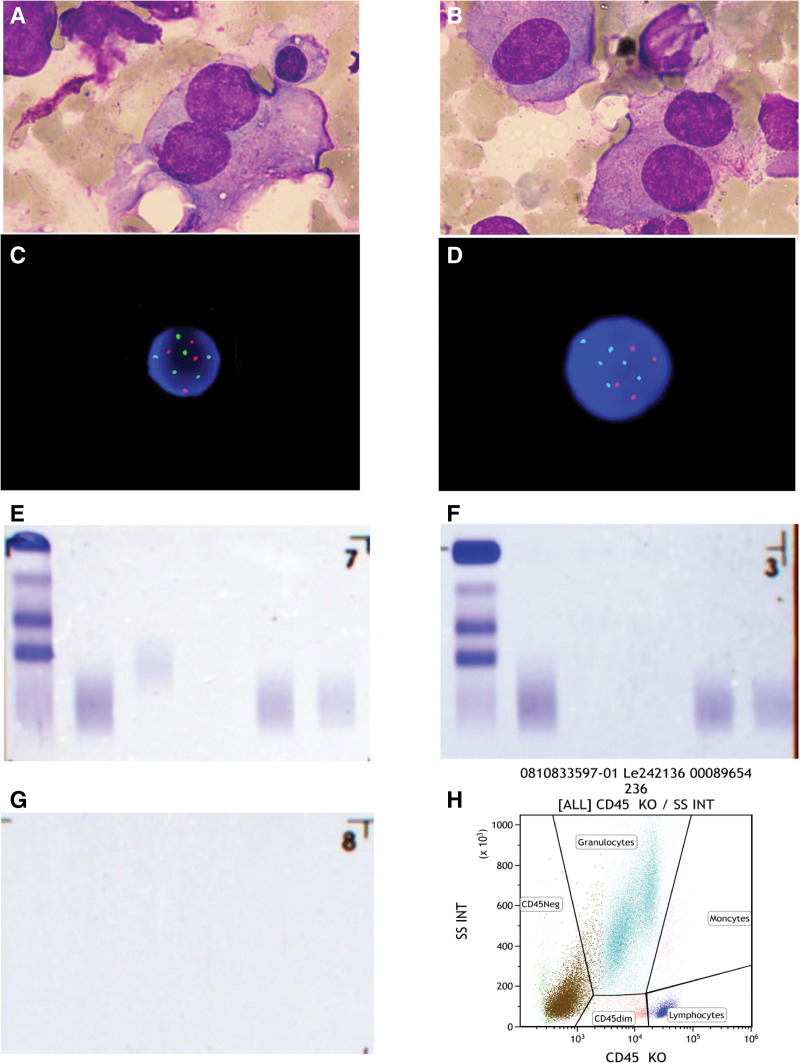
Paraclinical features of the patient. (A and B) Monoclonal plasma cells accounted for 17.5% of nuclear cells in bone marrow puncture smear. (C and D) FISH detected amplification of CKS1B (1q21) gene. (E) Immunofixation electrophoresis (IF). (F) Immunofixation electrophoresis (IgD + IgE). (G) Bence-Jones protein electrophoresis. (H) About 10.72% of monoclonal plasma cells were detected by FCM. FISH = fluorescence in situ hybridization.

The results of blood analysis are as follows (shown in Table 1). Low-dose whole-body bone CT showed multiple bone destruction of the skull, clavicle, scapula, sternum, ribs, multiple vertebrae and accessories of the spine, pelvis, bilateral humerus and proximal femur, multiple rib fractures, and multiple vertebral compression wedges, consistent with MM findings (shown in Fig. 1). No abnormal monoclonal bands were detected by hematuria immuno-fixation electrophoresis, and bence jones protein was negative. Monoclonal plasma cells accounted for 17.5% of nuclear cells in bone marrow puncture smear. About 10.72% of monoclonal plasma cells were detected by flow cytometry. Fluorescence in situ hybridization detected amplification of CKS1B (1q21) gene, t(4; 14), t(14; 16), t(14; 20), p53 mutations were negative (shown in Fig. 2).

**Table 1:** The results of blood analysis.

Blood examination before treatment	Patient results	Normal range
White blood cells	4.4	(3.5–9.5) × 10^9^/L
Red blood cells	2.53	(3.8–5.1) × 10^12^/L
Hemoglobin	76	(115–150) g/L
Platelet	180	(125–350) × 10^9^/L
Total protein	60.6	(65–85) g/L
Albumin	40.7	(40–55) g/L
Globulin	19.9	(20–40) g/L
Alanine aminotransferase	76	(0–40) U/L
Aspartate aminotransferase	11	(0–35) U/L
Alkaline phosphatase	188	(30–120) U/L
Gamma-glutamyltransferase	102	(7–45) U/L
Lactate dehydrogenase	356	(0–248) U/L
Glucose	6.47	(3.89–6.11) mmol/L
Urea	8.1	(2.8–7.6) mmol/L
Creatinine	107	(41–73) μmol/L
Uric acid	544	(154.7–357.0) μmol/L
Calcium	2.25	(2.20–2.65) mmol/L
β2-microglobulin	6	(0.8–2.4) mg/L
Erythrocyte sedimentation rate	67	(0–15) mm/h
Activated partial thromboplastin time	38.70	(25.1–37) S
Plasma fibrinogen	4.92	(2–4) g/L
Immunoglobulin IgG	4.03	(7–16) g/L
Immunoglobulin IgA	0.47	(0.7–4) g/L
Immunoglobulin IgM	0.05	(0.4–2.3) g/L

*Diagnosis*^[4]^: Multiple myeloma (non-secretory type) (DS Staging System: III A; ISS Staging System: III A; R-ISS Staging System: III A; mSMART High Risk). The patients received VCD chemotherapy (bortezomib 1.3 mg/m^2^, d1, d4, d8, d11; cyclophosphamide 300 mg/m^2^, d1–2, d8–9; dexamethasone sodium phosphate 20 mg, d1–2, d4–5, d8–9, d11–12, once every 21 days).

**Figure 3.**
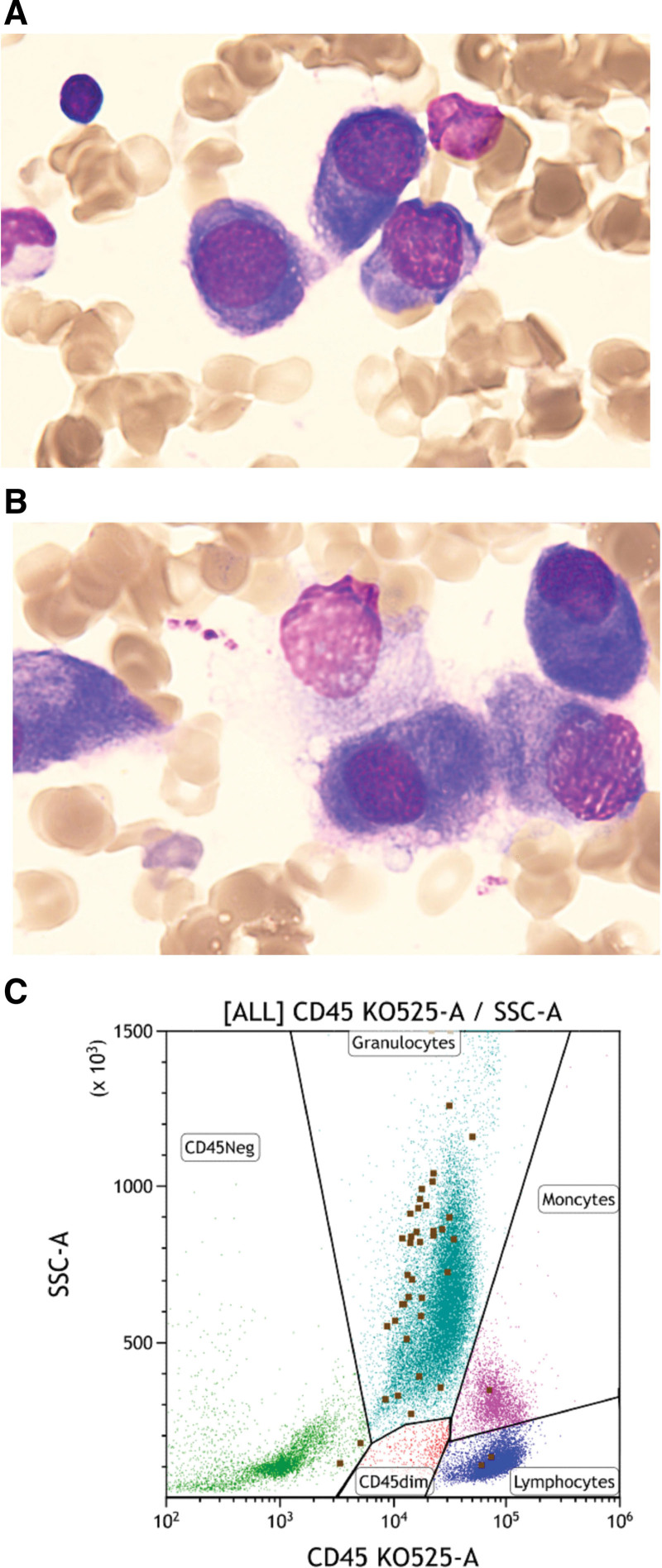
Paraclinical features of the patient after 2 cycles of VCD treatment. (A and B) 7.5% of monoclonal plasma cells were found on bone marrow smears. (C) 0.18% of monoclonal plasma cells were found by FLC. FLC=free light chain.

After 2 cycles of VCD treatment, the symptoms of bone pain were significantly relieved. We conducted an efficacy evaluation, and found that 7.5% of monoclonal plasma cells were found on bone marrow smears, and about 0.18% of monoclonal plasma cells were found by FLC (shown in Fig. 3), which was assessed as partial response.^[5]^ The patient is currently undergoing the fourth cycle of chemotherapy.

## 3. Discussion

The onset of MM is insidious, the median age of diagnosis is 69 (65–74) years,^[6]^ most of them are in the advanced stage of the disease at the time of treatment. Clinical symptoms are heterogeneous, usually showing “CRAB” symptoms. However, NSMM usually does not have “CRAB” symptoms, and the pathogenesis is not clear, which is related to the synthesis or secretion of immunoglobulins caused by a variety of factors.^[7]^ Clinical lack of specific detection indicators. When combined with other diseases, the symptoms have a high probability of overlapping, and more auxiliary examinations (such as bone and bone marrow examination) are needed as the basis for exclusion and diagnosis.

NSMM is characterized by multiple bone destruction on imaging. It is often caused by bone pain, which can occur in a single or multiple parts of the body, most commonly in the vertebral body, skull, ribs, pelvis, and proximal long bone.^[8,9]^ Multiple osteolytic lesions can be seen in the affected bones, causing pathological fractures, vertebral collapse, spinal cord compression, etc,^[10,11]^ which are easily misdiagnosed as osteoporosis, fracture, cervical and lumbar diseases, and bone metastatic cancer. As a rare disease in clinical practice, the misdiagnosis rate of NSMM is as high as 80%, among which bone pain ranks first among all misdiagnosed symptoms. At present, there are only a few minority studies and case reports, which do not attract enough attention from clinicians, and are more likely to miss diagnosis and misdiagnosis. Our patient started with low back pain and was admitted to the orthopedics and pain departments. He was diagnosed with osteoporosis, lumbar disc herniation, and lumbar disc fracture. Due to lack of correct diagnosis and treatment, condition worsened progressively over the past year and expanded to the whole back pain with positive sternal tenderness. He was then referred to the hematology department of our hospital and finally diagnosed with NSMM.

NSMM is consistent with other types of MM in treatment. It has been reported that the prognosis of NSMM is better than other types, which may be related to the metabolic advantage of immunoglobulin (because cells need to consume energy to synthesize or secrete immunoglobulin), or to the dependence of NSMM cells on proteasomal degradation of retained protein products, and they are more sensitive to proteasome-targeted drugs such as bortezomib.^[12,13]^ A number of retrospective studies have shown that the prognosis of NSMM under the treatment of immunomodulators combined with proteasome inhibitors is better than that of other subtypes, and the overall survival of NSMM is more prominent after hematopoietic stem cell transplantation.^[14,15]^ After receiving 2 cycles of VCD induction chemotherapy, hemoglobin increased, bone pain symptoms were significantly relieved, and the efficacy was evaluated as partial response. Follow-up chemotherapy will continue to be completed on schedule. In the follow-up treatment of the patient, autologous hematopoietic stem cell transplantation will be performed. We will continue to follow up to further evaluate the overall survival and progression-free survival.

## 4. Conclusion

NSMM is a rare disease without specific clinical symptoms and detection indicators, it is easy to be missed or misdiagnosed, which poses a challenge to the diagnosis of clinicians. For patients with no obvious cause of bone pain, poor treatment effect, and even progressive aggravation of bone pain symptoms and anemia, the possibility of MM should be considered. When the immunoglobulin IGA, IGg, and IGM are normal or reduced, the presence of NSMM should also be alerted.

## Acknowledgments

Thanks to the Heping Hospital affiliated to Changzhi Medical College for providing the necessary data.

## Author contributions

**Conceptualization:** Jie Li.

**Formal analysis:** Guoxiang Zhang.

**Resources:** Wenjie Zhang, Li Ge.

**Writing – review & editing:** Xi Li, Li Liang.

**Writing – original draft:** Qianshuang Geng.
